# Study on the performance of cement composites using composite-activated coal gangue

**DOI:** 10.1371/journal.pone.0355188

**Published:** 2026-08-06

**Authors:** Chaoyang Luo, Jing Bi

**Affiliations:** Department of Civil Engineering, Guizhou University, Guiyang, Guizhou, China; SASTRA Deemed University, INDIA

## Abstract

This study investigated the feasibility of utilizing thermally activated coal gangue as a partial replacement for Portland cement through a composite activation strategy involving red mud and sodium silicate. Orthogonal experimental design, together with isothermal calorimetry, low-field nuclear magnetic resonance (LF-NMR), X-ray diffraction (XRD), and scanning electron microscopy coupled with energy-dispersive spectroscopy (SEM–EDS), was employed to systematically evaluate the hydration behavior, microstructural evolution, and compressive strength development of the cementitious system. he results showed that sodium silicate significantly influenced hydration kinetics by delaying the main hydration stage and broadening the exothermic peak. LF-NMR analysis further indicated that systems containing sodium silicate maintained continuous water consumption during the later stages of hydration, suggesting a sustained hydration process. Red mud contributed to the regulation of hydration and partially compensated for the strength reduction associated with the incorporation of thermally activated coal gangue. Compared with sodium silicate activation alone, the combined incorporation of red mud and sodium silicate resulted in lower compressive strength at all curing ages. RD and SEM–EDS analyses indicated that the co-activated system exhibited a relatively heterogeneous distribution of hydration products and a less compact microstructure. These microstructural characteristics were consistent with its comparatively lower mechanical performance. Overall, this study provides insight into the interaction between red mud and sodium silicate in coal gangue-based cementitious materials and offers theoretical support for optimizing composite activation strategies and promoting the efficient utilization of industrial solid wastes.

## 1. Introduction

In 2024, China produced 4.78 billion tons of coal, accounting for 51.7% of global coal production [1]. Over the past four years, cumulative coal production reached 18.18 billion tons, representing approximately 66.6% of the country’s primary energy production, while coal consumption accounted for about 53% of total energy consumption [2]. These statistics indicate that coal is expected to remain the dominant energy source in China for the foreseeable future.

Coal gangue, a major byproduct of coal mining, accounts for approximately 15–20% of raw coal production, with annual generation reaching 150–200 million tons. The large-scale accumulation of coal gangue occupies substantial land resources [3,4] and poses potential risks to the ecological environment and human health [5–7]. Nevertheless, coal gangue contains abundant aluminosilicate minerals and valuable elements, providing considerable potential for resource utilization and environmentally sustainable disposal [8].

Among its mineral constituents, kaolinite can be transformed into highly reactive metakaolin through thermal activation. Owing to its high contents of reactive SiO_2_and Al_2_O_3_, thermally activated coal gangue exhibits pozzolanic activity and can participate in cement hydration reactions [9,10]. Consequently, it has attracted increasing attention as a supplementary cementitious material and a partial replacement for Portland cement [11].

The incorporation of thermally activated coal gangue into cement-based materials not only helps mitigate the environmental problems associated with its disposal but also reduces clinker consumption during cement production. As a result, it can lower production costs, promote the utilization of industrial solid waste, and decrease both energy consumption and carbon emissions in the cement industry [12–14].

To enhance the cementitious activity of coal gangue, researchers have developed various activation methods, including thermal activation [15,16], mechanical activation [17,18], chemical activation [19,20], and microwave activation [21]. Single activation methods, such as mechanical activation, thermal activation, and alkali activation, have been shown to improve the reactivity of coal gangue and enhance material properties, including compressive strength, impermeability, and microstructural characteristics [22–25]. Compared with single-treatment approaches, composite activation strategies can further promote the release of reactive SiO_2_ and Al_2_O_3_, thereby improving pozzolanic activity, mechanical performance, and overall reactivity [26–31].

Among the available activation methods, thermal activation is considered one of the most practical and widely adopted approaches. Previous studies have reported that the optimal calcination temperature for coal gangue generally ranges from 700 to 800 °C [30,32,33]. Within this temperature range, kaolinite undergoes substantial dehydroxylation and is transformed into highly reactive metakaolin, maximizing the availability of reactive SiO_2_ and Al_2_O_3_. This promotes subsequent hydration reactions and facilitates the formation of hydration products such as C–S–H gel, resulting in enhanced pozzolanic activity. The optimal calcination duration is typically around 2h. However, excessive calcination temperatures may adversely affect the reactivity of coal gangue. When the temperature exceeds 800 °C, particularly above 950 °C, crystalline phases such as mullite and spinel begin to form. The formation of these phases reduces the content of amorphous materials and reactive components, thereby decreasing pozzolanic activity and diminishing the effectiveness of thermal activation [34]. As a result, thermally activated coal gangue has been successfully applied in cement-based materials [35], geopolymers [36], soil stabilization, and coal gangue-based bricks [37]. In addition to thermal activation, alkali activation has also been widely used to enhance the reactivity of coal gangue, as it promotes the dissolution of aluminosilicate phases and the formation of cementitious products. Building on this, combined thermal and alkali activation has been recognized as a more effective strategy, where thermal treatment increases the amount of reactive amorphous phases, while alkali activation further facilitates their dissolution and reaction, leading to improved overall performance.

Although previous studies have demonstrated that thermal activation and alkali activation can effectively enhance the reactivity of coal gangue, studies on composite cementitious systems involving coal gangue, red mud, and sodium silicate are still limited. In particular, the interaction among these components and their roles in governing hydration behavior remain insufficiently understood. The synergistic effects on hydration kinetics, microstructural evolution, and strength development in such multi-component systems have not been systematically established. To address this gap, this study investigates thermally activated coal gangue activated individually and jointly by red mud and sodium silicate. The hydration process is characterized using isothermal calorimetry and low-field nuclear magnetic resonance (LF-NMR), while the hydration products, microstructure, and mechanical properties are analyzed using XRD, SEM-EDS, and compressive strength tests. By correlating hydration kinetics, pore structure evolution, and mechanical performance, this study aims to elucidate the synergistic activation mechanism of coal gangue-based composite cementitious systems.

## 2. Materials and methods

### 2.1. Raw materials

#### 2.1.1. Sources.

The coal gangue used in this study was obtained from Guizhou Province, while ordinary Portland cement (Grade P·O 42.5) and sintered red mud (procured from Yantai, Shandong Province) were used as received. Tap water was used throughout all experiments. The main chemical compositions of coal gangue, red mud, and cement are summarized in Table 1. An industrial sodium silicate solution was used as the alkali activator, with a SiO_2_ content of 29.8%, Na_2_O content of 13.2%, a silicate modulus (SiO_2_/Na_2_O molar ratio) of 2.4, and a solid content of 50%

**Table 1:** Main chemical compositions of cementitious materials (wt.%).

Raw Material	CaO	SiO_2_	Al_2_O_3_	MgO	Fe_2_O_3_	K_2_O
Cement	68.89	23.15	2.96	0.85	0.30	0.22
Coal gangue	1.51	63.59	23.19	0.13	5.94	3.66
Red mud	46.85	21.23	5.50	1.15	7.59	2.03

#### 2.1.2. Process.

**Fig 1:**
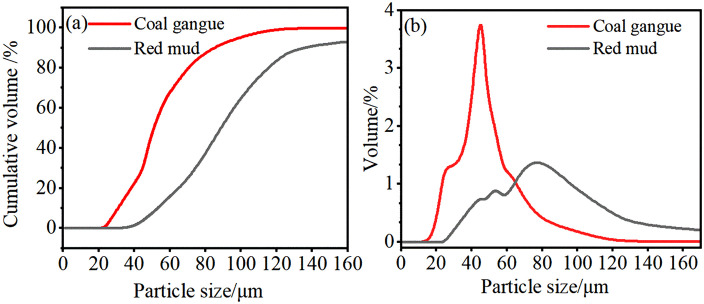
Particle size distribution curves of coal gangue and red mud. (a) Cumulative particle size distribution. (b) Volume-based particle size distribution.

The raw coal gangue was first crushed and coarsely pulverized, followed by fine grinding in a ball mill. Particles passing through a 120μm sieve were collected by vibratory sieving. Based on previous studies and the work of Zhang et al. [38], the optimal thermal activation conditions for coal gangue were determined to be calcination at 700 °C for 2h in a muffle furnace, with a heating rate of 10 °C/min. After calcination, the material was allowed to cool naturally to room temperature. The red mud was oven-dried at 105 °C until a constant mass was achieved, and particles finer than 150μm were obtained by sieving. The particle size distribution of coal gangue is presented in Fig 1.

### 2.2. Test Methods

#### Isothermal Calorimetry (IC).

Isothermal calorimetry was performed using a TA Instruments TAM III isothermal calorimeter (USA) to continuously record the heat evolution of hydration for 72 h at 23 °C. All raw materials were equilibrated at 23 °C prior to testing. A baseline was recorded for 30–60 min before each measurement to ensure instrument stability. The paste was prepared by high-speed mixing for 60 s and then transferred into the sample ampoule within 2 min.

#### Low-Field Nuclear Magnetic Resonance (LF-NMR) Testing.

Low-field nuclear magnetic resonance (LF-NMR) measurements were conducted using a MesoMR23-060H-I NMR analyzer (Nimag Corporation, China) operating at a resonance frequency of 12MHz. The transverse relaxation time (T_2_) was measured using the Carr–Purcell–Meiboom–Gill (CPMG) pulse sequence. The acquisition parameters were as follows: echo time (TE) = 100μs, number of echoes = 2000, 90° and 180° pulse widths of 6.52μs and 12.48μs, respectively, sampling frequency = 250 kHz, analog gain = 20.0, and digital gain = 3.

#### Compressive Strength Test.

Compressive strength was measured using a DSZ-1000 multi-field coupling testing machine under displacement-controlled mode at a loading rate of 0.1 mm/min. Three specimens were tested for each mix, and the average value was reported.

#### X-Ray Diffraction (XRD) Analysis.

X-ray diffraction (XRD) analysis was performed using a Rigaku Ultima IV diffractometer with Cu Kα radiation (λ = 1.5418 Å), operated at 40 kV and 40 mA. Measurements were conducted over a 2θ range of 10°–80° with a step size of 0.02°.

#### Scanning Electron Microscopy and Energy-Dispersive X-Ray Spectroscopy (SEM-EDS).

Microstructural morphology was examined using a ZEISS Sigma 360 field-emission scanning electron microscope (Germany). Elemental analysis of selected micro-regions was performed using an integrated energy-dispersive X-ray spectroscopy (EDS) system. Measurement points were selected based on morphological features observed in SEM images.

### 2.3. Test Mix Proportion

To evaluate the activation effect of calcined coal gangue under different alkali activation systems, including red mud, sodium silicate, and their combination, an orthogonal experimental design was employed. Three influencing factors were selected, namely red mud content, sodium silicate modulus, and water-to-binder ratio (W/B), each at three levels. An L₉(3⁴) orthogonal array was adopted for the experimental design [39], as shown in Table 2.

**Table 2:** Factors and levels of the orthogonal experiment.

level	Red mud (%)	Sodium silicate modulus	W/B	error
	(A)	(B)	(C)	(D)
1	10	1.2	0.42	1
2	30	1.6	0.45	2
3	50	2.0	0.48	3

Based on the results of the orthogonal experiments, the optimal red mud replacement ratio in the coal gangue–red mud blend, sodium silicate modulus, and water-to-binder ratio (W/B) were determined to be 30%, 2.0, and 0.45, respectively. For the mechanistic investigation, the total replacement level of supplementary cementitious materials was fixed at 30% of the binder. Accordingly, the optimized red mud proportion corresponds to a mixture containing 9% red mud and 21% calcined coal gangue. Representative groups were then prepared under these optimized conditions to investigate the individual and combined activation effects of red mud and sodium silicate, as summarized in Table 3. The sample IDs are defined as follows: OP denotes the plain Portland cement control; CG denotes the coal gangue–cement binary group; CGS represents the coal gangue–cement group activated with sodium silicate; CGR denotes the coal gangue–red mud–cement ternary group without sodium silicate; and CGRS represents the coal gangue–red mud–cement group activated with sodium silicate.

**Table 3:** Mix proportions of experimental groups.

Sample ID	Cement (%)	Coal gangue (%)	Red mud (%)	Water glass (%)	W/B
OP	100	0	0	0	0.45
CG	70	30	0	0	0.45
CGS	70	30	0	3	0.435
CGR	70	21	9	0	0.45
CGRS	70	21	9	3	0.435

Note: All contents are expressed as percentages of the total binder mass. W/B represents the total water-to-binder ratio. For CGS and CGRS, part of the mixing water was supplied by the sodium silicate solution; therefore, the externally added water was adjusted to 0.435 to maintain a constant total W/B of 0.45.

### 2.4. Specimen preparation

Prismatic specimens with dimensions of 40 mm × 40 mm × 160 mm were prepared in accordance with GB/T 17671−2021 (equivalent to the ISO standard method for determining cement mortar strength). Nine experimental groups were included in the orthogonal design. For each group, three replicate specimens were prepared and tested at each curing age. The fluidity of the fresh systems was measured prior to casting. After molding, the specimens were cured under standard curing conditions and tested at curing ages of 3, 7, and 28 days. Compressive strength was determined using a DSZ-1000 multi-field coupling testing machine. For microstructural characterization, samples were collected from the interior of the specimens at the designated curing ages. To terminate the hydration process, the samples were immediately immersed in anhydrous ethanol for 48 h. Subsequently, they were dried in a vacuum oven at 40 °C for 8 h and sealed until further analysis. For LF-NMR measurements, fresh paste was poured into a custom-made PVC cylindrical mold (Φ25 mm × 50 mm). The mold was then sealed and placed in the NMR probe. Data acquisition commenced 15 min after the initial contact between water and the binder.

### 2.5. Flowchart

**Fig 2:**
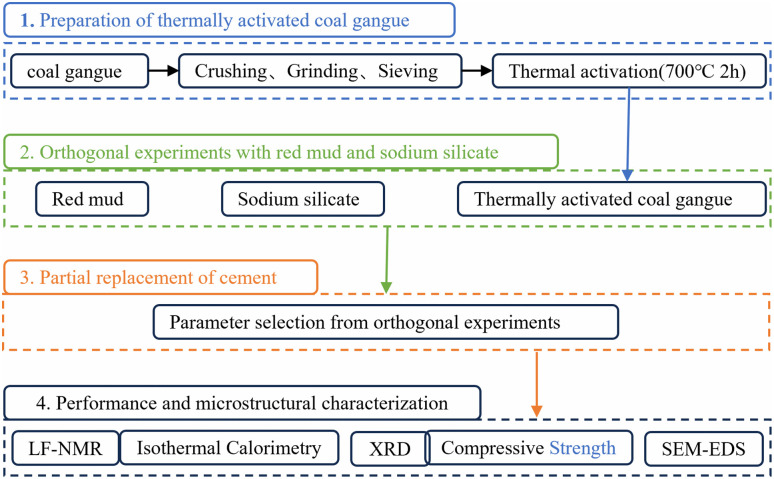
Test flowchart.

The overall research workflow is presented in Fig 2 and consists of four stages. First, raw coal gangue was crushed, ground, sieved, and thermally activated to obtain thermally activated coal gangue. Second, orthogonal experiments incorporating red mud and sodium silicate were conducted to determine the optimal activation conditions and mix proportions. Third, the optimized composite-activated coal gangue system was used as a partial replacement for cement. Finally, the hydration behavior, hydration kinetics, compressive strength, and microstructural evolution of the prepared specimens were investigated using isothermal calorimetry (IC), low-field nuclear magnetic resonance (LF-NMR), X-ray diffraction (XRD), and SEM–EDS.

## 3. Results and discussion

### 3.1. Raw Material XRD Analysis

**Fig 3:**
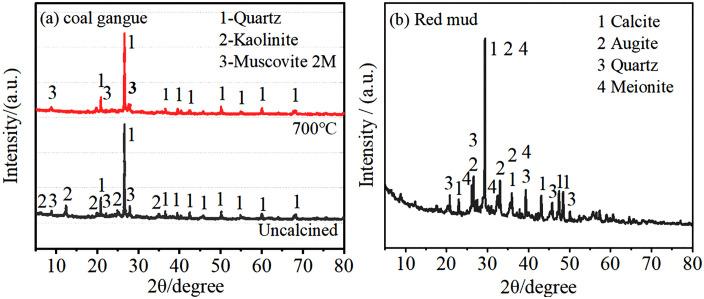
X-ray diffraction (XRD) patterns of coal gangue and red mud. (a) XRD patterns of coal gangue. (b) XRD pattern of red mud.

The mineralogical compositions of coal gangue, thermally activated coal gangue, and red mud were characterized by X-ray diffraction (XRD), and the corresponding patterns are presented in Fig 3. The raw coal gangue primarily consisted of kaolinite, quartz, and a small amount of muscovite. After thermal activation at 700°C for 2h, the characteristic diffraction peak of kaolinite at 2θ = 12.38° nearly disappeared, indicating the destruction of its crystalline structure due to dehydroxylation. This result is consistent with the formation of a more disordered aluminosilicate phase, which is generally associated with enhanced pozzolanic reactivity. In contrast, the diffraction peak of quartz at 2θ = 26.6° remained prominent after calcination, reflecting its relatively high thermal stability. A slight reduction in peak intensity was observed, which may be related to a decrease in crystallinity during thermal treatment [40]. It is noteworthy that the kaolinite peaks in the raw coal gangue were relatively weak even before calcination. This phenomenon may be partially attributed to the crushing and ball-milling processes employed during sample preparation, which can reduce the crystallinity of kaolinite through mechanical activation [41]. The red mud exhibited a more complex mineralogical composition, mainly comprising calcite, quartz, pyroxene, and calcium silicate phases.

### 3.2. Orthogonal Experiment

The results of the orthogonal experiment are listed in Table 4. Each compressive strength value is expressed as the mean ± standard deviation of three replicate specimens (n = 3). The relatively small standard deviations indicate acceptable repeatability and reliability of the experimental measurements. Range analysis was conducted to visually determine the primary and secondary order of influence of each factor on the fluidity and compressive strength of the composite-activated coal gangue at different ages. Following this, an Analysis of Variance (ANOVA) was performed. The F-value for each factor was calculated and compared with the critical F-value at a given significance level to assess the statistical significance of their effects.

**Table 4:** Orthogonal experimental results for the combined activation of coal gangue.

Group	Factors			Fluidity (mm)	Compressive strength (MPa)		
	A	B	C		3d	7d	28d
1	10	1.2	0.42	168	8.3 ± 0.39	12.8 ± 1.10	22.7 ± 1.50
2	10	1.6	0.45	194	12.2 ± 0.58	16.6 ± 1.35	24.8 ± 1.75
3	10	2.0	0.48	212	7.5 ± 0.63	9.1 ± 1.47	20.6 ± 1.80
4	30	1.2	0.45	182	9.3 ± 0.95	17.3 ± 1.65	25.9 ± 1.71
5	30	1.6	0.48	210	10.7 ± 0.82	21.1 ± 1.78	22.2 ± 1.49
6	30	2.0	0.42	172	13.8 ± 0.64	23.8 ± 1.62	32.6 ± 1.32
7	50	1.2	0.48	198	5.7 ± 0.96	7.4 ± 1.55	12.5 ± 1.75
8	50	1.6	0.42	165	8.0 ± 0.70	20.0 ± 1.58	27.3 ± 1.85
9	50	2.0	0.45	188	10.2 ± 0.56	15.4 ± 1.34	29.1 ± 1.84

Note: Values of compressive strength are expressed as mean ± standard deviation (n = 3).

#### 3.2.1. Range analysis.

Considering the standard deviations reported in Table 4, the variability among replicate specimens was generally much smaller than the differences observed between experimental groups, indicating good experimental repeatability and confirming the reliability of the range analysis. Range analysis was conducted based on the results of the orthogonal experiment, and the corresponding results are summarized in Table 5. This analysis was used to evaluate the relative influence of the three factors—red mud content (A), sodium silicate modulus (B), and water-to-binder ratio (W/B, C)—on the performance of the composite-activated coal gangue system and to rank their significance for each performance indicator.

**Table 5:** Range analysis of the influencing factors.

Factors	A	B	C	D(error)	Note
k1	191.33	182.67	168.33	188.67	Fluidity
k2	188.00	189.67	188.00	188.00	
k3	183.67	190.67	206.67	186.33	
R	7.66	8.00	38.34	2.34	
k1	9.33	7.77	10.03	9.73	3-Day Compressive strength
k2	11.27	10.30	10.57	10.57	
k3	7.97	10.50	7.97	8.27	
R	3.30	2.73	2.60	2.30	
k1	12.83	12.93	18.87	16.43	7-Day Compressive strength
k2	20.73	19.23	16.43	16.37	
k3	14.70	16.10	12.97	15.47	
R	7.90	6.30	5.90	0.96	
k1	22.70	22.03	27.53	24.67	28-Day Compressive strength
k2	26.90	24.77	26.60	24.97	
k3	24.63	27.43	20.10	24.60	
R	4.20	5.40	7.43	0.37	

For the fresh paste fluidity, the order of influence of the three factors was W/B (C)> sodium silicate modulus (B)> red mud content (A). For the 3 d and 7 d compressive strengths, the factors exhibited the same order of influence, namely red mud content (A)> sodium silicate modulus (B) > W/B (C). In contrast, the 28 d compressive strength was most strongly influenced by W/B (C), followed by sodium silicate modulus (B) and red mud content (A). These results indicate that the dominant factors governing the properties of the composite-activated coal gangue system vary with curing age.

**Fig 4:**
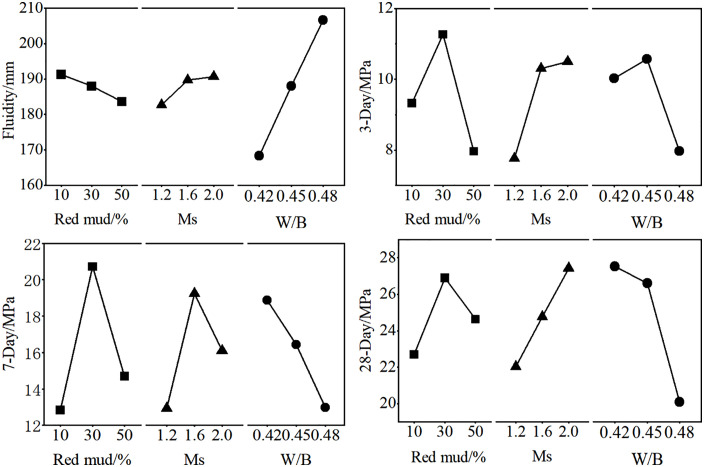
Effects of the experimental factors on fluidity and compressive strength.

The effects of the three experimental factors (where Ms denotes the sodium silicate modulus) on the fluidity and compressive strength of the composite-activated coal gangue system are presented in Fig 4. Fluidity initially increased with increasing red mud content (A), reached a maximum at the intermediate level, and then decreased slightly. Increasing the sodium silicate modulus (B) and W/B (C) both improved the fluidity, with W/B exerting the strongest influence. The effects of the three factors on compressive strength were strongly dependent on the curing age. The early-age compressive strength (3d and 7d) was primarily governed by the red mud content (A), whereas the 28d compressive strength was most sensitive to W/B (C). This demonstrates that the dominant factors controlling strength development change as hydration progresses.

Moreover, the relatively small standard deviations of the compressive strength measurements further confirm the good repeatability of the experimental results and support the reliability of the subsequent optimization analysis.

#### 3.2.2. Analysis of Variance.

To further evaluate the statistical significance of the experimental factors and quantify the contribution of experimental error, an analysis of variance (ANOVA) was performed for all performance indicators. The corresponding results are summarized in Tables 6–9. This analysis complements the range analysis by providing a statistical basis for assessing the significance of each factor on the measured properties. The significance levels were determined according to the following criteria: an F-value greater than F₀.₀₁(2,2) indicated a highly significant effect (***), an F-value between F₀.₀₅(2,2) and F₀.₀₁(2,2) indicated a moderately significant effect (**), and an F-value between F₀.₁(2,2) and F₀.₀₅(2,2) indicated a generally significant effect (*). An F-value lower than F₀.₁(2,2) was considered statistically insignificant.

**Table 6:** ANOVA results for fluidity.

Factor	S	f	S/f	F-value	Critical F-value	Significance
A	88.67	2	44.34	10.23	F_0.1_ = 9.00 F_0.05_ = 19.00 F_0.01_ = 99.00	*
B	114.00	2	57.00	13.15		*
C	2204.67	2	1102.34	254.38		***
D	8.67	2	4.19			

**Table 7:** ANOVA results for 3 d compressive strength.

Factor	S	f	S/f	F-value	Critical F-value	Significance
A	16.50	2	8.25	2.03	F_0.1_ = 9.00 F_0.05_ = 19.00 F_0.01_ = 99.00	
B	13.93	2	6.96	1.71		
C	11.32	2	5.66	1.39		
D	8.14	2	4.07			

**Table 8:** ANOVA results for 7 d compressive strength.

Factor	S	f	S/f	F-value	Critical F-value	Significance
A	102.30	2	51.15	58.49	F_0.1_ = 9.00 F_0.05_ = 19.00 F_0.01_ = 99.00	**
B	59.64	2	29.77	34.04		**
C	52.75	2	26.37	30.16		**
D	1.75	2	0.87			

**Table 9:** ANOVA results for 28 d compressive strength.

Factor	S	f	S/f	F-value	Critical F-value	Significance	
A	26.52	2	13.26	115.84	F_0.1_ = 9.00 F_0.05_ = 19.00 F_0.01_ = 99.00		***
B	43.74	2	21.87	191.11			***
C	98.38	2	49.19	429.80			***
D	0.23	2	0.12				

For fluidity, the F-value of the water-to-binder ratio (C) reached 254.38, which was substantially higher than F_0.01_(2,2) =99.00, indicating a highly significant effect. In comparison, the F-values of red mud content (A) and sodium silicate modulus (B) were 10.23 and 13.15, respectively, both exceeding F₀.₁(2,2) =9.00, indicating generally significant effects on fluidity. The relatively small error sum of squares (8.67) suggests that experimental variability was well controlled, confirming the reliability of the measurements.

The statistical significance of compressive strength varied with curing age. At 3 d, all F-values were below F₀.₁(2,2) =9.00, indicating no statistically significant effects of the investigated factors. Although the compressive strength results exhibited good repeatability, as reflected by the relatively small standard deviations reported in Table 4, the differences among experimental groups were comparable to the residual variation. As a result, the factor effects at this curing age were not sufficiently distinguishable from experimental noise to achieve statistical significance. This behavior is typical of early-age hydration, where the development of mechanical properties has not yet fully progressed.

At 7d, the F-values of all factors fell between F_0.05_(2,2) and F_0.01_(2,2), indicating moderately significant effects and a clear increase in the influence of the experimental factors compared with the 3 d results. By 28 d, all F-values exceeded F_0.01_(2,2), demonstrating highly significant effects of all three factors. Meanwhile, the error sum of squares remained considerably lower than those of the factor terms, further confirming the robustness and reliability of the statistical analysis at later curing ages.

Overall, the ANOVA results are consistent with the range analysis and demonstrate that the influence of red mud content, sodium silicate modulus, and water-to-binder ratio becomes progressively more significant with increasing curing age.

#### 3.2.3. Construction of a comprehensive evaluation function.

To comprehensively evaluate the overall performance of the alkali-activated red mud–coal gangue system, a single performance indicator is insufficient to reflect the differences among different mix proportions. Therefore, based on the results of the orthogonal experiments, flowability and compressive strength at different curing ages were integrated using the Analytic Hierarchy Process (AHP) to establish a multi-criteria comprehensive evaluation model. Range analysis and analysis of variance (ANOVA) were further employed to support the optimization of the experimental scheme.

(1) Establishment of the evaluation index system. Flowability (F), 3 d compressive strength (S_3_), 7 d compressive strength (S_7_), and 28 d compressive strength (S_28_) were selected as the evaluation indicators.
(2) Construction of the judgment matrix. According to the principle of prioritizing mechanical performance while considering workability, and taking into account the different contributions of strength development at various curing ages, the 1–9 scale method was used for pairwise comparisons. The resulting judgment matrix is:

$$\begin{array}{c} \left[\begin{array}{cccc} \text{1} & \text{1} & \text{1}/\text{2} & \text{1}/\text{3}\\ \text{1} & \text{1} & \text{1} & \text{1}/\text{2}\\ \text{2} & \text{1} & \text{1} & \text{1}/\text{2}\\ \text{3} & \text{2} & \text{2} & \text{1} \end{array}\right] \end{array}$$

(3) Weight calculation. The judgment matrix was normalized by columns, and the average value of each row was used to derive the weight vector. The calculated weights are listed in Table 10. The results indicate that 28 d compressive strength contributes most significantly to the comprehensive evaluation, followed by 7 d and 3 d compressive strengths, while flowability has the lowest weight.

**Table 10:** Weights of evaluation indicators obtained by AHP.

Indicator	Weight
F	0.16
$S_{\text{3}}$	0.20
$S_{\text{7}}$	0.25
$S_{\text{28}}$	0.39

(4) Consistency check. A consistency test was conducted to verify the reliability of the judgment matrix. The consistency index (CI) and consistency ratio (CR) were calculated, and the results showed that CR < 0.1, indicating that the judgment matrix has acceptable consistency and the derived weights are reliable.

#### Comprehensive evaluation function.

To eliminate the influence of different dimensions, all performance indicators were normalized prior to analysis. Based on the AHP-derived weights, the comprehensive evaluation function was established as:


$$\begin{array}{c} Y=\text{0}.\text{16}F+\text{0}.\text{20}S_{\text{3}}+{\text{0}.\text{25}S}_{\text{7}}+{\text{0}.\text{39}S}_{\text{28}} \end{array}$$

where Y represents the comprehensive performance index; F is the normalized flowability; and S_3_, S_7_, and S_28_ are the normalized compressive strengths at different curing ages.

Taking Y as the response variable, range analysis was performed, and the results are summarized in Table 11.

**Table 11:** Range analysis of the comprehensive evaluation index (Y).

Factor	A	B	C	D(Error)
K1	1.20	0.93	1.64	1.53
K2	2.02	1.73	1.73	1.60
K3	1.25	1.80	1.09	1.34
k1	0.40	0.31	0.55	0.51
k2	0.67	0.58	0.58	0.53
k3	0.42	0.60	0.36	0.45
R	0.27	0.29	0.21	0.09

The results indicate that the influence of factors on the comprehensive performance follows the order: B > A > C > D. The optimal combination is identified as B_3_A_2_C_2_D_2_.

To further assess the statistical significance of each factor on the comprehensive performance, ANOVA was conducted. The results are summarized in Table 12.

**Table 12:** ANOVA of the comprehensive evaluation index (Y).

Factor	S	df	S/f	F-value	F-critical value	Significance
Red mud	0.14	2	0.07	11.45	F_0.1_ = 9.00 F_0.05_ = 19.00 F_0.01_ = 99.00	*
Modulus	0.16	2	0.08	12.67		*
W/B	0.08	2	0.04	6.57		
Error	0.01	2	0.01			

The results show that sodium silicate modulus has a significant effect on the overall performance, while red mud content exhibits a moderate effect. The influence of the water-to-binder ratio is relatively weak. These findings are consistent with those obtained from range analysis.

Based on the comprehensive evaluation model combined with range analysis and ANOVA, the optimal mix proportion of the alkali-activated coal gangue system was determined as 30% red mud content, sodium silicate modulus of 2.0, and a water-to-binder ratio of 0.45. Under these conditions, the system achieves a balanced performance in both workability and mechanical properties, providing a quantitative basis for optimizing the utilization of coal gangue-based cementitious materials.

### 3.3. Heat of Hydration

**Fig 5:**
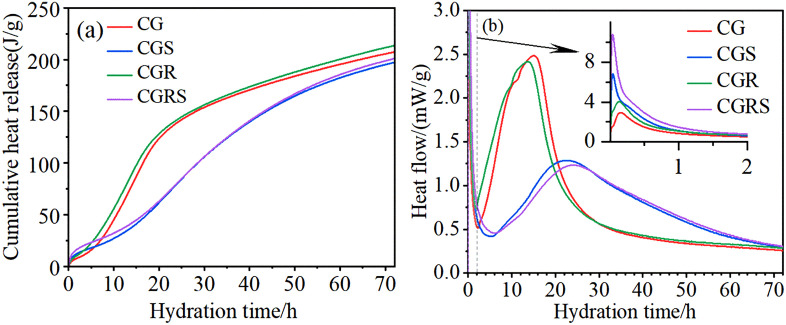
Cumulative heat release (a) and heat release rate (b) over 72 hours.

The cumulative hydration heat and heat flow rate curves of the CG, CGR, CGS, and CGRS over 72 h are presented in Fig 5. All pastes exhibited an initial exothermic peak within approximately 0.2h, corresponding to the rapid dissolution of reactive phases and initial wetting of the binder particles.

The CGS and CGRS containing sodium silicate showed a higher initial heat release rate compared with the CG and CGR. This behavior is associated with the increased alkalinity introduced by sodium silicate, which promotes the dissolution of aluminosilicate species at the early stage. For the CG and CGR, the main exothermic peaks appeared at approximately 15h and 14h, respectively. During the acceleration period, the CGR exhibited a higher cumulative heat release and a higher heat flow rate than the CG, with an earlier occurrence of the main hydration peak. This indicates that the presence of red mud contributes to enhanced early reactivity, which may be attributed to its alkaline components and fine particles providing additional nucleation sites for hydration products, thereby facilitating the early hydration process.

In contrast, the CGS and CGRS exhibited delayed main exothermic peaks at approximately 23h and 24h, respectively, accompanied by a noticeable reduction in peak intensity. The main peak of the CGS appeared slightly earlier than that of the CGRS. These results suggest that sodium silicate modifies the hydration kinetics by delaying the main hydration peak and reducing the peak heat release rate, indicating a retarding effect on the early hydration process under the present experimental conditions.

### 3.4. LF-NMR

#### 3.4.1. Evolution of Water Relaxation Characteristics.

The T_2_ spectra of the samples were obtained by inverting the NMR decay signals [42–44]. Changes in the transverse relaxation signals were monitored to characterize the evolution of water during the hydration process. In the T_2_ spectrum, relaxation times shorter than 0.01ms correspond to chemically bound water, whereas signals in the ranges of 0.01–1ms and 1–100ms are assigned to water in gel pores and capillary pores, respectively. Owing to the detection limit of the instrument, signals below 0.01ms could not be directly detected. Therefore, the reduction in the detectable T_2_ signal was regarded as the conversion of free water into chemically bound water. To quantitatively characterize the evolution of water during hydration, the weighted average transverse relaxation time (T_2W_) was calculated according to Equation (1) [45,46]:


$$\begin{array}{c} T_{2W}=\frac{\underset{\text{0}}{\overset{T_{\text{2}}}{\int }}T_{\text{2}}I_{T_{\text{2}}}{dlgT}_{\text{2}}}{\underset{\text{0}}{\overset{T_{\text{2}}}{\int }}I_{T_{\text{2}}}d{lgT}_{\text{2}}} \end{array}$$ (1)

**Fig 6:**
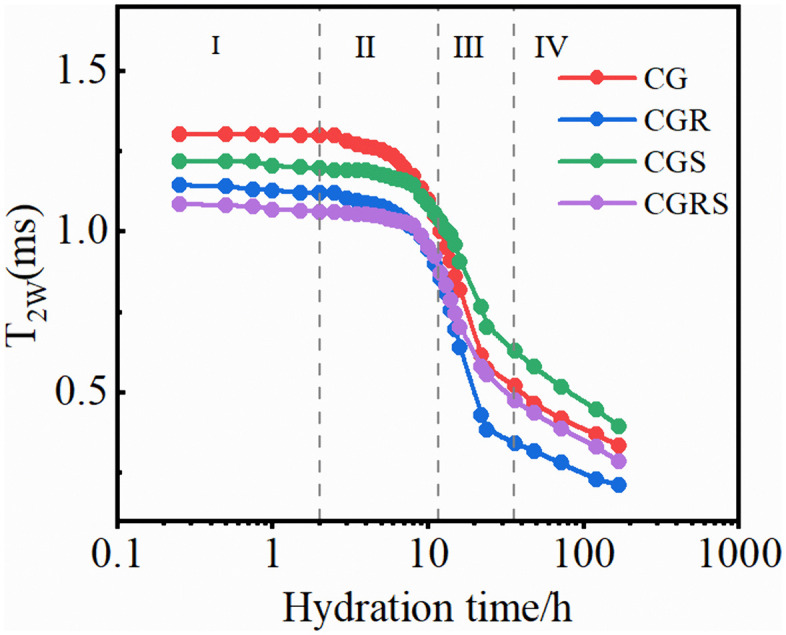
Decay curve of the weighted average T2W relaxation time.

Where $T_{2W}$ denotes the weighted average $T_{\text{2}}$ value; $T_{\text{2}}$ and $I_{T_{\text{2}}}$ represent the transverse relaxation time and corresponding relaxation intensity, respectively. The calculation results are shown in Fig 6.

To further identify the transition points between different hydration periods, the second derivative of the T_2W_ evolution curve with respect to hydration time was calculated. The zero-crossing points of the second derivative were used to determine the boundaries between successive hydration periods. Based on this method, the hydration process was divided into four periods: the Induction period, Acceleration period, Deceleration period, and Stabilization period. The transition times and durations of each hydration period are summarized in Table 13.

**Table 13:** Transition times and durations of hydration periods.

	Sample	CG	CGR	CGS	CGRS
Hydration time	t_1_	2.01	1.86	3.65	3.59
	t_2_	11.56	11.32	15.58	15.32
	t_3_	35.68	36.21	46.32	46.25
Duration of each period	Induction period	2.01	1.86	3.65	3.59
	Acceleration period	9.55	9.46	11.93	11.73
	Deceleration period	24.12	24.89	30.74	30.93
	stabilization period	–	–	–	–

Induction period. As summarized in Table 13, the incorporation of red mud slightly shortened the induction period from 2.01 h for CG to 1.86 h for CGR, indicating a modest acceleration of the initial hydration process. In contrast, the addition of sodium silicate markedly prolonged the induction period to 3.65 h for CGS, representing an increase of approximately 81% relative to CG. The induction period of CGRS (3.59 h) was almost identical to that of CGS, demonstrating that the retarding effect of sodium silicate dominated the initial hydration process, while the contribution of red mud remained limited. This observation is consistent with the delayed occurrence of the main exothermic peak revealed by isothermal calorimetry.

Acceleration period. The duration of the acceleration period changed only slightly after the incorporation of red mud, decreasing from 9.55 h for CG to 9.46 h for CGR. By comparison, sodium silicate significantly prolonged this period to 11.93 h for CGS, corresponding to an increase of approximately 25%. A similar duration (11.73 h) was observed for CGRS, indicating that sodium silicate governed the hydration kinetics during this period, whereas red mud exerted only a minor modifying effect.

Deceleration period. A similar trend was observed during the deceleration period. The incorporation of red mud produced only a slight increase in duration from 24.12 h to 24.89 h. In contrast, sodium silicate prolonged the deceleration period to approximately 31 h, corresponding to an increase of about 28% compared with CG. The nearly identical durations of CGS and CGRS further confirm that sodium silicate remained the dominant factor controlling the later hydration process.

Stabilization period. The onset of the stabilization period was delayed from 35.68 h for CG to 46.32 h for CGS, representing a delay of approximately 10.6 h. The stabilization period of CGRS began at 46.25 h, differing from that of CGS by only 0.07 h, which further demonstrates that sodium silicate dominated the overall hydration process, whereas the addition of red mud produced only a marginal adjustment.

Overall, red mud exerted only a limited influence on the durations of the individual hydration periods. In contrast, sodium silicate substantially prolonged the induction, acceleration, and deceleration periods and consequently delayed the onset of the stabilization period. The hydration behavior of CGRS closely resembled that of CGS, indicating that sodium silicate played the dominant role in regulating the hydration kinetics, while red mud mainly provided a slight promoting effect during the early stage of hydration.

#### 3.4.2. Hydration degree.

This study uses the relaxation signal intensity of low-field nuclear magnetic resonance to evaluate the hydration evolution of the groups, and its expression is shown in Equation (2) [47]:


$$\begin{array}{c} \alpha_{1\text{f}}=\frac{I_{\text{0}}-I_{T}}{I_{\text{0}}}\times \frac{X}{\gamma } \end{array}$$ (2)

where I₀ represents the relaxation signal quantity at the beginning of hydration, I_T_ indicates the relaxation signal quantity at time t, X represents the actual water-to-binder ratio used, and γ represents the theoretical water demand required for complete hydration. In this study, γ was calculated based on the Powers hydration model and was determined to be 0.42. LF-NMR was used to monitor the evolution of water states during hydration. The LF-NMR results were used for comparative analysis among different groups.

**Fig 7:**
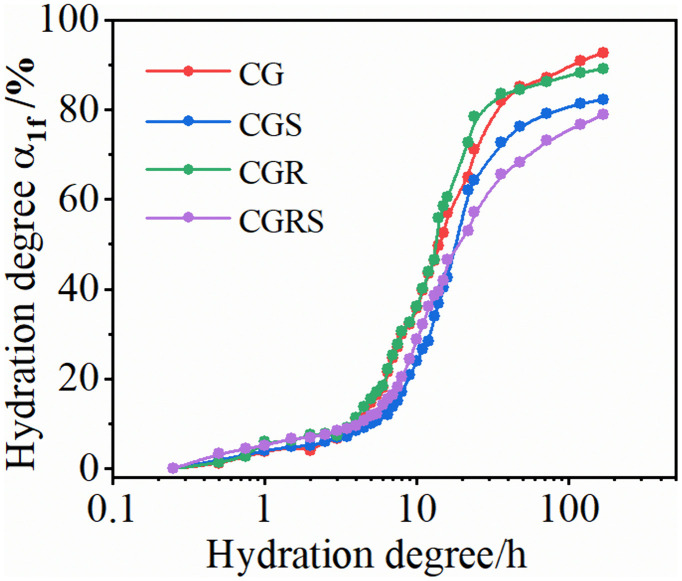
The variation characteristics of hydration degree over time.

As shown in Fig 7, the incorporation of red mud and sodium silicate affects the hydration evolution of the system. Compared with the CG group, all modified groups exhibit a reduced calculated hydration degree throughout the hydration process. Among them, sodium silicate shows a more pronounced inhibitory effect than red mud. The combined incorporation of red mud and sodium silicate results in the lowest calculated hydration degree among all groups. This behavior suggests that alkali-related components significantly alter the water consumption and hydration kinetics of the system.

### 3.5. Compressive Strength

**Fig 8:**
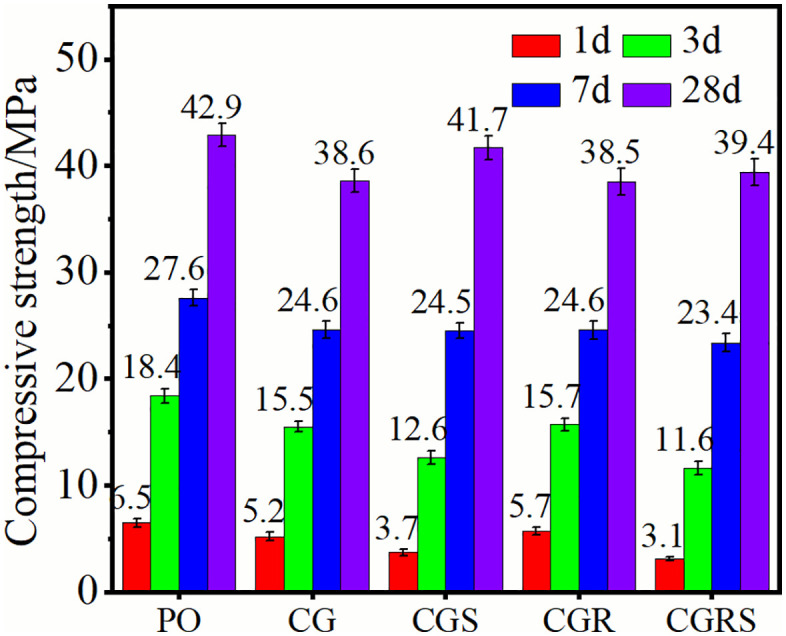
Compressive strengths of different paste systems at various curing ages (mean ± SD).

The compressive strengths of the different paste groups at various curing ages are presented in Fig 8. Each value represents the mean of three parallel specimens (mean ± SD, n = 3), and the error bars indicate the corresponding standard deviations. The relatively small standard deviations demonstrate good repeatability of the compressive strength measurements.

Compared with PO, replacing 30% of the cement with thermally activated coal gangue (CG) reduced the compressive strength at all curing ages. Nevertheless, the 28-day compressive strength of CG reached approximately 90% of that of PO, indicating that thermally activated coal gangue retained considerable cementitious activity after thermal activation. Replacing part of the thermally activated coal gangue with red mud (CGR) resulted in compressive strengths comparable to those of CG throughout the curing period. This finding suggests that the incorporation of red mud partially compensated for the strength reduction through the combined effects of reactive calcium phases and micro-filling. A different strength development pattern was observed for CGS. Compared with CG, CGS exhibited lower compressive strength at early ages, whereas its strength increased more rapidly during later curing. At 28 days, the compressive strength of CGS exceeded that of CG and reached approximately 97% of that of PO. Combined with the hydration kinetics results presented in Sections 3.3 and 3.4, these results indicate that sodium silicate delayed early hydration while promoting subsequent development of the cementitious matrix, thereby improving later-age compressive strength.

In contrast, CGRS consistently exhibited lower compressive strength than CGS throughout the curing period. This result indicates that the incorporation of red mud did not further enhance the activation effect of sodium silicate under the present mixture proportions. Instead, the coexistence of red mud and sodium silicate may have modified the hydration process and the formation of hydration products, resulting in a less compact microstructure than that developed in CGS. The underlying mechanisms are further discussed based on the XRD and SEM–EDS analyses in the following sections.

### 3.6. Analysis of Hydration Products and Microstructure

#### 3.6.1. XRD Analysis of Hydration Products.

**Fig 9:**
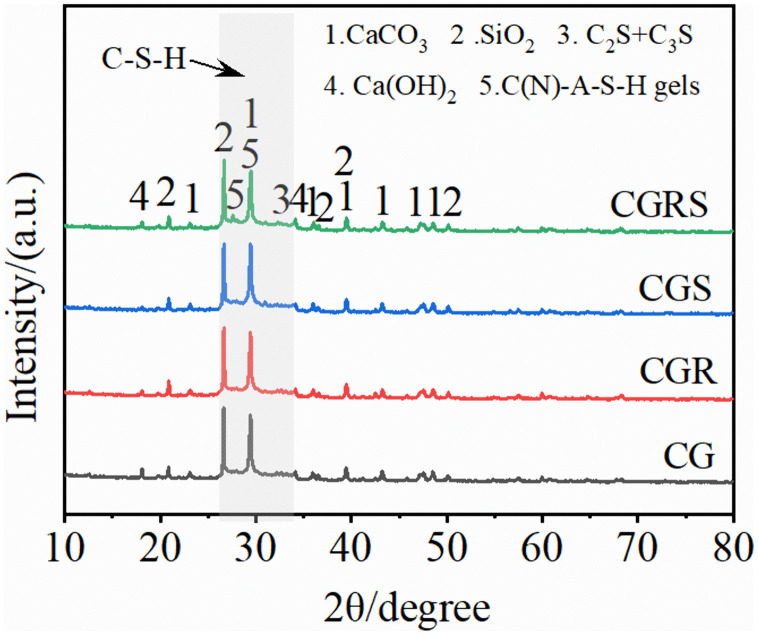
XRD of 28-day hydration products.

The XRD patterns of the hydrated pastes at 28 days are presented in Fig 9. In CG, characteristic diffraction peaks of calcium hydroxide (CH) were observed at approximately 2θ = 18.0° and 34.1°. A strong diffraction peak at 2θ = 26.6° was assigned to quartz originating from thermally activated coal gangue, while weak reflections corresponding to residual clinker phases (C_3_S and C_2_S) were also detected. In addition, a broad diffuse hump appeared within the range of approximately 25–35°, which is generally attributed to poorly crystalline hydration products, primarily C–S–H gel [48]. Because this diffuse hump overlaps with crystalline phases such as quartz and CH, it was used only for qualitative interpretation rather than quantitative analysis of the amorphous phase. Compared with CG, CGR exhibited weaker CH diffraction peaks, indicating a lower residual CH content at 28 days. Meanwhile, the diffuse hump in the 25–35° region became slightly more pronounced. These observations suggest that the incorporation of red mud influenced the evolution of the hydration products and promoted the formation of additional poorly crystalline phases. For CGS, the CH diffraction peaks were further reduced, and the reflections corresponding to residual C_3_S and C_2_S became less distinct than those observed for CG. At the same time, the diffuse hump within the 25–35° range became more prominent. These changes indicate that the addition of sodium silicate modified the hydration process and increased the amount of poorly crystalline reaction products.

Among the four groups, CGRS exhibited relatively weak CH diffraction peaks together with a more pronounced diffuse hump in the 25–35° region. These observations suggest that the combined incorporation of red mud and sodium silicate further modified the hydration products and increased the proportion of poorly crystalline phases. These findings are further supported by the SEM–EDS analysis presented in the following section.

#### 3.6.2. SEM-EDS analysis and Image-Based Pore Area Evaluation.

**Fig 10:**
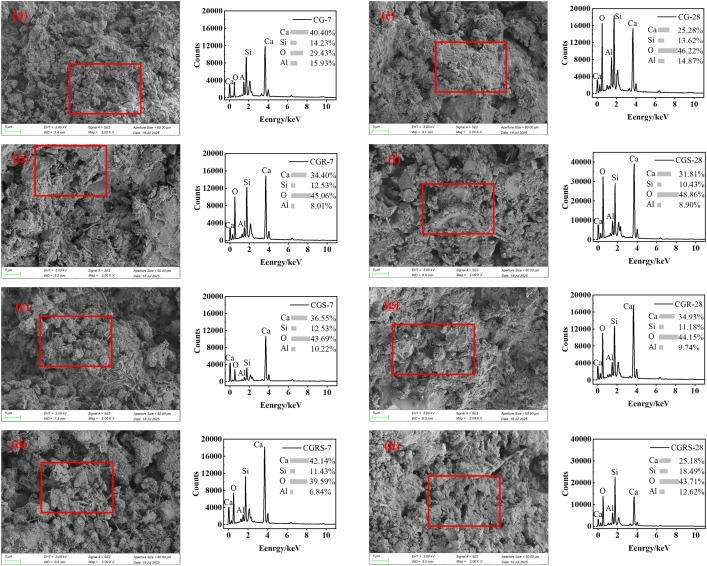
SEM-EDS analysis of hydration product.

The microstructure and elemental composition of the hydration products were characterized using Scanning Electron Microscopy (SEM) coupled with Energy Dispersive X-ray Spectroscopy (EDS). Representative microstructures of the four groups at 7 and 28 days are shown in Fig 10, while the corresponding EDS results for the selected regions are presented alongside the SEM images.

At 7 days, both CG and CGR exhibited typical early-age hydration characteristics of cement-based materials. In CG, hydration products gradually covered the surfaces of coal gangue particles, while the blurred particle boundaries suggested the initiation of pozzolanic reactions. In CGR, the incorporation of red mud increased the Al content in the hydration products. The Si/Al ratio of the representative gel region increased from 0.89 in CG to 1.56 in CGR, suggesting enhanced incorporation of Al into the hydration products and the possible formation of Al-modified calcium silicate hydrate phases. The addition of sodium silicate significantly altered the evolution of the microstructure. In the CGS group, a larger amount of gel-like hydration products was observed at 7 days, filling the spaces between particles and forming a more continuous matrix. This indicates that sodium silicate promoted the formation of hydration products and accelerated the development of the microstructure. In contrast, although hydration products were also observed in the CGRS system, their spatial distribution appeared less uniform, with locally agglomerated gel phases and discontinuous coverage of some particle surfaces.

At 28 days, all systems exhibited further development of hydration products and a more compact microstructure. The CG formed a relatively continuous C–S–H gel matrix, effectively binding coal gangue particles and refining pore space. A similar densification trend was observed in the CGR, indicating that the incorporation of red mud did not hinder the long-term hydration process. Among all specimens, CGS exhibited the most homogeneous and compact microstructure. Hydration products were more uniformly distributed, and visible pores were substantially reduced. In comparison, the CGRS still exhibited locally loose regions and partially connected pore structures, indicating a relatively higher degree of microstructural heterogeneity.

To provide supplementary quantitative information regarding microstructural compactness, image-based pore area analysis was performed on representative SEM images using ImageJ software. After grayscale conversion and threshold segmentation, the apparent pore area fraction was calculated as the ratio of segmented dark areas to the total analyzed area. It should be noted that this analysis was intended for comparative evaluation among different systems rather than absolute porosity quantification.

The calculated apparent pore area fractions are summarized in Table 14, showing a consistent decrease with curing age for all systems, which indicates progressive hydration and microstructural densification.

**Table 14:** Apparent pore area fraction of different systems obtained from SEM image analysis.

ID	Age	Pore area fraction (%)	Age	Pore area fraction (%)
CG	7d	12.95	28d	9.80
CGR	7d	13.12	28d	10.42
CGS	7d	9.33	28d	6.33
CGRS	7d	15.09	28d	10.72

These quantitative results are consistent with the SEM observations. The lower apparent pore area fraction of CGS suggests that sodium silicate promoted the formation of a more continuous and compact gel network, thereby refining pore space and improving microstructural connectivity. In contrast, the relatively higher apparent pore area fraction observed in CGRS indicates a less uniform distribution of hydration products and a more heterogeneous microstructure. The image-based pore analysis therefore provides additional support for the superior mechanical performance of CGS and the relatively lower strength of CGRS.

Overall, the SEM–EDS observations together with the image-based pore area analysis reveal a clear structure–performance relationship. CG exhibiting a more continuous gel network and lower apparent pore area fraction developed higher compressive strength, whereas increased microstructural heterogeneity was associated with comparatively lower mechanical performance.

## 4. Conclusions

This study systematically investigated the combined activation effects of red mud and sodium silicate on thermally activated coal gangue in cement-based composite systems. The results clarify the distinct roles of red mud and sodium silicate in regulating hydration and strength development, providing new insight into the activation mechanism of thermally activated coal gangue. Based on these findings, the main conclusions are summarized as follows:

(1) Sodium silicate significantly regulates the hydration kinetics of the composite system. It delays early hydration, reduces the peak heat release rate, and broadens the main exothermic peak, thereby extending the duration of the main hydration stage. LF-NMR results further indicate that both CGS and CGRS maintain continuous pore water consumption at later ages, suggesting a sustained hydration process.
(2) The strength development strongly depends on the activation strategy. Red mud partially compensates for the strength reduction caused by cement replacement, whereas CGS suppresses early-age strength but significantly enhances later-age strength, achieving approximately 97% of the 28-day compressive strength of the Portland cement control. In contrast, CGRS remains weaker than CGS throughout the curing period.
(3) XRD and SEM–EDS analyses show that sodium silicate promotes the formation of a denser hydration-product network, whereas CGRS exhibits a relatively heterogeneous microstructure with localized porous regions, consistent with its comparatively lower compressive strength.

## Supporting information

S1 File Dataset. (7Z)
